# Orthogonal alignment of multilayered MC3T3-E1 cells induced by cyclic stretch

**DOI:** 10.1007/s10237-025-01978-z

**Published:** 2025-07-02

**Authors:** Shuichiro Suzuki, Ken Imajo, Junfeng Wang, Jeonghyun Kim, Eijiro Maeda, Kazuaki Nagayama, Takeo Matsumoto

**Affiliations:** 1 Biomechanics Laboratory, Department of Mechanical Systems Engineering, Graduate School of Engineering, Nagoya University, Furo‑cho, Chikusa‑ku, Nagoya, Aichi 464‑8603 Japan; 2 Biomechanics Laboratory, Department of Mechanical Engineering, Nagoya Institute of Technology, Gokiso-cho, Showa-ku, Nagoya, Aichi 466-8555 Japan; 3 Micro-Nano Biomechanics Laboratory, Department of Mechanical Systems Engineering, Ibaraki University, Nakanarusawa-cho, Hitachi, Ibaraki 316-8511 Japan

**Keywords:** Cell biomechanics, Mechanobiology, Cyclic stretch, Orientation, Multilayered cells, MC3T3-E1

## Abstract

When cyclic stretch is applied to a monolayer of cells cultured on an elastic substrate, many types of cells align in the direction perpendicular to the stretch or along the direction of minimal substrate strain. However, the behavior of multilayer cells under cyclic stretch remains unclear. In this study, we cultured MC3T3-E1 osteoblast-like cells at high density to form multilayer cells and subjected them to cyclic stretch with an amplitude of 10% at 1 Hz. We found that the lower layer cells aligned in the direction of the stretch after 12 h, whereas the upper layer cells aligned perpendicular to the direction of stretch after 24 h. The 10% cyclic stretch was transmitted to the upper layer cells as approximately 5% at the onset of the stretch and increased over time, reaching 7% at 12 h when the lower layer cells completed alignment in the direction of stretch. This suggests that sufficient cyclic stretch transmitted to the upper layer led to the alignment of the upper layer cells in the perpendicular direction after 12 h. On the other hand, reducing intracellular tension with Y-27632 caused cells in both upper and lower layers to align in the direction of stretch. In contrast, increasing intracellular tension with calyculin A eliminated significant alignment in both layers. These findings indicate that cell alignment is closely related to intracellular tension and that the alignment of the lower layer cells in the direction of stretch may be due to a decrease in intracellular tension.

**Supplementary Information:**

The online version contains supplementary material available at 10.1007/s10237-025-01978-z.

## Introduction

It is well known that cell monolayer cultured on a substrate shows orientation in response to various external mechanical stimuli. For example, when fluid shear stress is applied to confluent endothelial cells, they elongate and align in the direction of flow (Nerem et al. 1981; Ookawa et al. 1992). In contrast, such a response is not observed under sparse conditions (Shaka et al. 2024). Furthermore, cells adhered to elastic substrates exhibit various responses when subjected to cyclic stretch. For instance, cells are often reported to align in the direction of minimal strain of the substrate (Wang et al. 1995, 2001; Takemasa et al. 1998; Nagayama et al. 2012; Livne et al. 2014) or in the direction perpendicular to the stretch (Takemoto et al. 2023). On the other hand, when macrophages are subjected to cyclic stretch, they align in the direction of stretch (Matsumoto et al. 1996), and it has also been reported that the orientation of cells varies depending on the type of cyclic waveform applied to the cells (Nagayama et al. 2012; Tondon and Kaunas, 2012). Additionally, the effect of substrate stiffness has been noted: Cells on a stiff substrate align in a direction perpendicular to the cyclic stretch, whereas cells on a soft substrate align in the direction of the stretch (Sekar et al., 2023; Tondon et al., 2014). The influence of cell tension has also been highlighted, where reducing the tension of cells that typically align perpendicular to the direction of stretch results in alignment in the direction of the stretch (Kaunas et al. 2005; Lee et al. 2010).

In general, organisms form tissues by clustering cells together to perform their functions. Furthermore, tissues often function by overlapping and orienting various cells in specific directions. Therefore, it is easy to imagine that the orientation of these tissues is affected by tissue deformation. Nevertheless, most of the previous research has been limited to studies applying cyclic stretch to monolayered cells or cells encapsulated in the extracellular matrix (Bono et al. 2016; Nagayama et al. 2009; Nieponice et al. 2007). To the best of the authors' knowledge, there has been little research on the behavior of cells under cyclic stretch in more physiologically relevant conditions, such as multilayered cells. Therefore, in this study, we focused on MC3T3-E1 cells, which are osteoblast-like cells, and investigated their behavior under cyclic stretch after layering the cells. As a result, we found that after applying cyclic stretch for 24 h, the cells in the lower layer aligned in the direction of the stretch, while the cells in the upper layers aligned in the direction perpendicular to the stretch. To investigate the underlying causes of these responses, we examined how much substrate deformation is transmitted through the multilayered cells. We also explored the differences in alignment when the tension in the stress fibers (SFs) was manipulated pharmacologically. Additionally, we observed the microstructure of the SFs and vinculins, a focal adhesion (FA) protein, to study the involvement of the SFs in the reorientation of cells in the upper and lower layer of cells.

## Materials & methods

### Cell culture

Mouse osteoblast-like MC3T3-E1 cells were obtained from the RIKEN BioResource Center (Japan). The cells were cultured in α-MEM (Wako, Japan) supplemented with 10% fetal bovine serum (FBS, Gibco, Japan) and 1% penicillin–streptomycin (Sigma, USA) in an incubator maintained at 37 °C with 5% CO_2_-95% air and 100% humidity. The medium was changed every 2 to 3 days. The MC3T3-E1 cells used in the experiments were passaged between 10 and 30 times after purchase.

### Fabrication of the stretch chamber

To apply cyclic stretch, we utilized the stretch chambers (STB-CH-04, Strex, Japan). The bottom membranes were fabricated in the laboratory using PDMS. The PDMS mixture was prepared by combining the base material Silpot 184 base (Dow Corning, USA) and the curing agent Silpot 184 cat (Dow Corning) in a 10:1 weight ratio. The mixture was degassed by allowing it to stand in a refrigerator for 30 min. Next, 1 mg of the mixture was dropped onto a 90-mm cell culture dish (Iwaki, Japan) and spread thinly using a spin coater (Aiden, Japan) at 500 rpm for 1 min, achieving a thickness of approximately 100 µm. The dish was then heated in an oven at 65 °C for 4 h. Afterward, the PDMS membrane was peeled from the dish and attached to the stretch chamber frame using the PDMS mixture to avoid wrinkles. The assembly was dried at 65 °C for 4 h. Excess material was trimmed, and the chamber was cleaned using ultrasonic cleaning with distilled water. The chamber was then sterilized by autoclaving at 121 °C and approximately 0.2 MPa pressure for 20 min. To enhance cell adhesion, the chamber was coated for 1 h with a solution prepared by mixing bovine plasma fibronectin (Sigma) and PBS(-) (Wako) in a 1:20 ratio. After each experiment, the used chambers were carefully cleaned with a cotton swab and alkaline detergent (Clean Ace, As One, Japan), followed by ultrasonic cleaning and autoclaving for reuse. The bottom membrane was replaced approximately every 5 experiments.

### Preparation of monolayered and multilayered cell samples

Monolayer cell samples were obtained by seeding cells at a density of 1 × 10^3^ cells/mm^2^ on the fibronectin-coated bottom of the chamber and culturing statically for 48 h to form a quasi-confluent monolayer. The multilayer cell samples were created by seeding a second layer of cells at a density of 2 × 10^2^ cells/mm^2^ on top of the monolayer sample and culturing statically for 24 h. This procedure resulted in a nearly complete multilayer cell sample covering the entire chamber.

### Application of cyclic stretch

The stretch chamber with either a monolayer or multilayer of cells seeded on the bottom was mounted on a cell stretching system (NS-350C, Scholar Tec Corp, Japan) and subjected to cyclic stretch. A triangular waveform with a frequency of 1 Hz and a strain amplitude of 10% was used to apply cyclic stretch to the cells. The experiments were conducted in the same incubator in which the cells were cultured. Additionally, the strain in the membrane on the bottom of the chamber was measured under the triangular waveform. It was found that the cyclic stretch in the direction of stretch was approximately 8–9%, while compressive strain in the direction perpendicular to the direction of stretch was about 4–4.5% (Fig. S1, Table S1). Based on these results, the direction of minimum strain was determined to be approximately 58–60°, with the direction of stretch defined as 0° (Table S2).

### Manipulation of intracellular tension

To decrease intracellular tension, the ROCK-specific inhibitor Y-27632 (Wako) was used. Y-27632 (10 μM) was added to the culture medium immediately prior to stretching. To increase intracellular tension, the phosphatase inhibitor calyculin A (Wako) was employed. Calyculin A was added to the culture medium at the concentrations of 0.25, 0.5, 0.75, 1, and 2 nM immediately prior to stretching.

### Strain measurement of multilayered cells

The deformation of the elastic substrate and its effect on each layer of cells were analyzed by examining changes in the distance between cell nuclei. To achieve this, cell nuclei were stained with Hoechst 33,342 (Invitrogen, USA) diluted at 1:10,000 in α-MEM for 10 min in an incubator. After staining, the samples were washed twice with PBS at 37 °C and then placed in α-MEM. The stretch chamber was mounted on the stage of a confocal laser scanning microscope (IX-81 + FV-1200, Olympus, Japan), subjected to 10% stretch, and images of nuclei in both the upper and lower layers were captured at the same positions before and after stretching. The experiment was conducted at room temperature. The distances between cell nuclei were measured using ImageJ (National Institutes of Health, USA). Specifically, two adjacent nuclei located either parallel or perpendicular to the direction of stretch were selected, and the distance between their centroids was measured three times (Fig. S2). The average of these measurements was used to calculate the strain on the cells resulting from a 10% deformation of the chamber, using the following equation:

1 $$\begin{array}{c}Strain \left(\%\right)=({l}_{10\%}/{l}_{0\%}-1)\times 100,\end{array}$$

where ${l}_{0\%}$ represents the distance between nuclei before stretching, and ${l}_{10\%}$ represents the distance between nuclei after 10% stretching.

### Immunofluorescence staining

The nucleus, actin filaments (AF), and vinculin were stained. Cells were fixed by soaking them in 10% formalin for 5 min. The cell membrane was permeabilized by treating the samples with 0.5% Triton-X for 10 min. For vinculin staining, the samples were blocked by soaking them in a 0.5% BSA/PBS(-) solution prepared by diluting bovine serum albumin (BSA, Sigma) in PBS(-) and incubating for 30 min at room temperature. After blocking, anti-vinculin antibody, clone V284 (Millipore, USA), diluted 1:50 in PBS(-), was added to the samples and incubated for 60 min at room temperature. Subsequently, a secondary antibody, rabbit anti-mouse IgG (H + L) (A11059, Molecular Probes, USA), diluted 1:100 in BSA/PBS(-), was added and incubated for 60 min at room temperature. For AF staining, Alexa Fluor 546 Phalloidin diluted 1:40 in PBS(-) was added to the samples and incubated for 60 min at room temperature. The nuclei were stained by adding Hoechst 33,342 diluted 1:1000 in PBS(-) to the samples and incubating for 10 min at room temperature. The stained samples were observed using a confocal laser scanning microscope (FV1200, Olympus, Japan, or LSM880, Zeiss, Germany).

### Orientation analysis and perpendicular orientation index

Since the alignment direction of AF generally corresponds to the orientation of cells, many studies have determined cell orientation based on AF alignment. In this study, we also analyzed the alignment of AF and used this direction as the orientation of the cells. To calculate the distribution of AF alignment angles relative to the direction of stretch, fluorescence images of AF (512 × 512 pixels) were analyzed using two-dimensional Fourier transformation with MATLAB (MathWorks, USA). The direction of stretch was set as 0°, and distributions were obtained in 10° increments counterclockwise. To determine whether the cells in each layer predominantly aligned in the direction of stretch or in the direction perpendicular to it, the perpendicular orientation index ${R}_{A}$ was defined by the following equation:

2 $$\begin{array}{c}{R}_{A}=\frac{\text{Orientation ratio of }90^\circ \pm 10^\circ }{\text{Orientation ratio of }0^\circ \pm 10^\circ }\end{array}$$

This index indicates that the cells are aligned in a direction perpendicular to the direction of stretch when* R*_*A*_ > 1.80, while when *R*_*A*_ < 1/1.80≒0.56, the cells are aligned in the direction of stretch. The value 1.80 was chosen because the *R*_*A*_ was approximately 1.8 when MC3T3-E1 cell monolayer was cyclically stretched for 12 h.

### Data analysis

The data are expressed as the mean ± SD. Significant differences was evaluated using the Student's t test. A value of *P* < 0.05 was considered significant for all analyses.

## Results

### Changes in orientations of multilayered cells in response to cyclic stretch

**Fig. 1:**
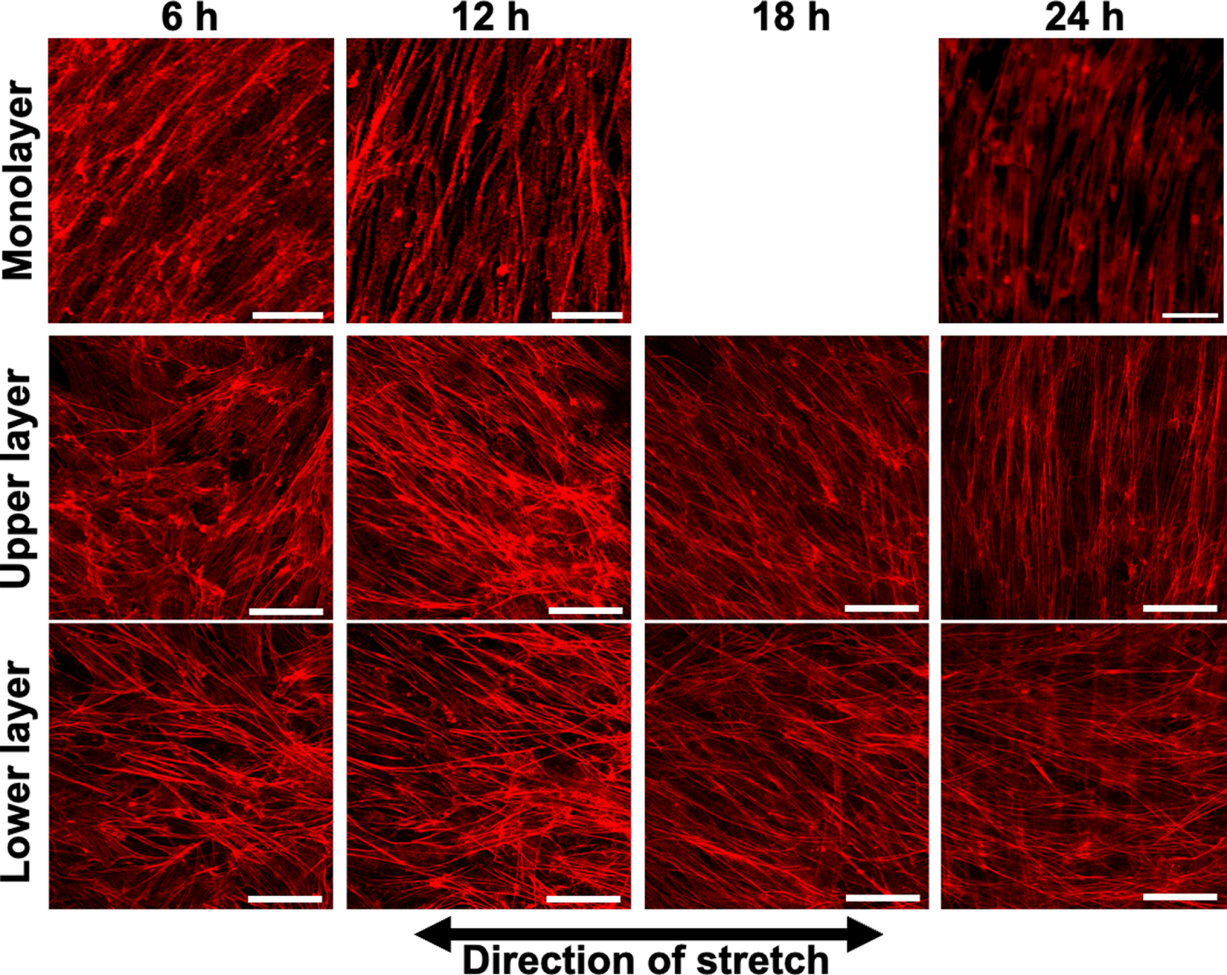
Example of the fluorescent images of actin filaments in monolayer and multilayer MC3T3-E1 cells exposed to cyclic stretch. All images were taken using a confocal laser scanning microscope, except for the monolayer at 24 h, which was captured using an inverted microscope and at a different site than those at 6 and 12 h. Bars = 50 µm

**Fig. 2:**
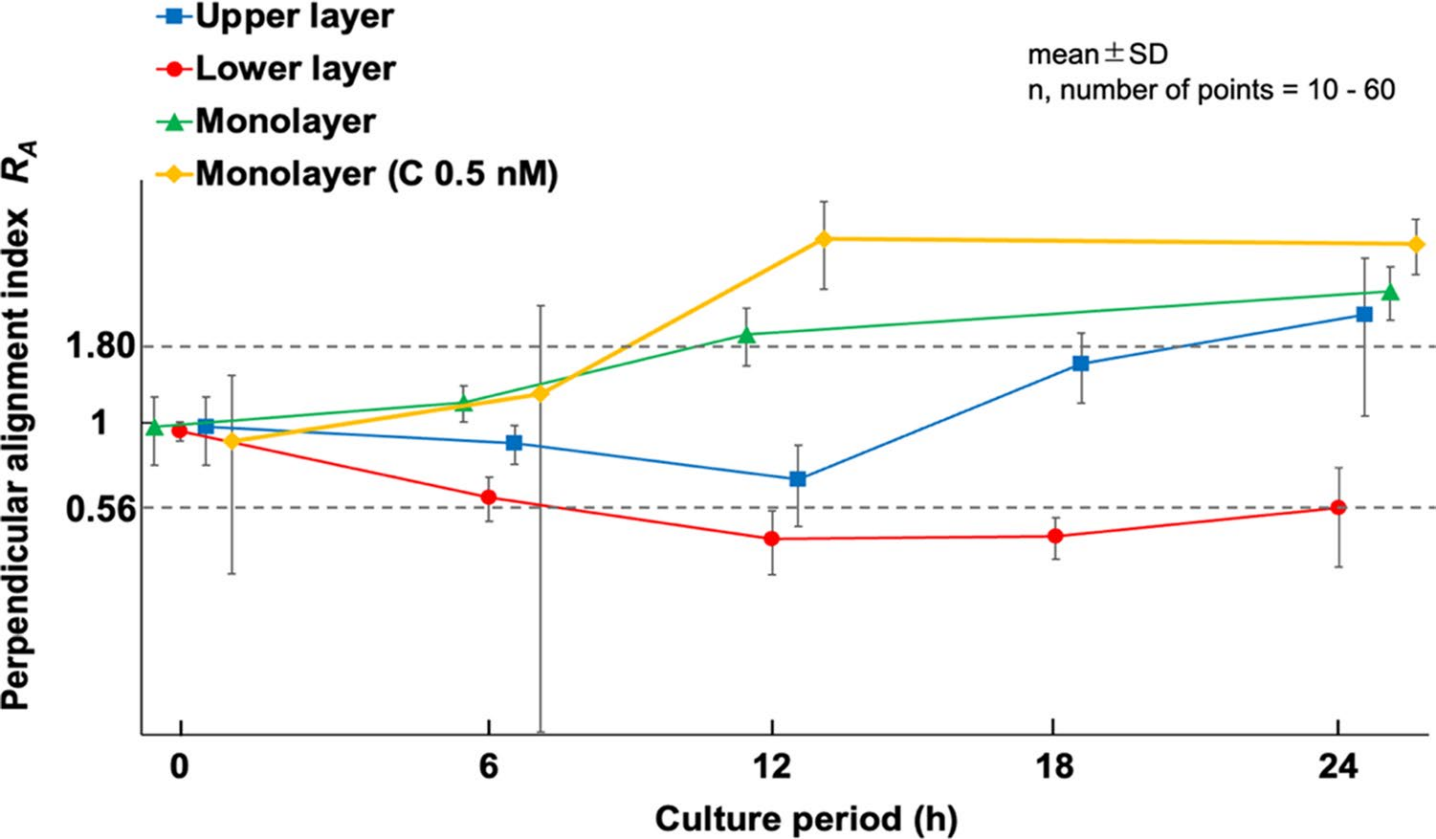
Time course changes of the perpendicular alignment index (*R*_*A*_) in multilayer MC3T3-E1 cells exposed to cyclic stretch. C, calyculin A

An example of fluorescence images of AF in the upper and lower layers of MC3T3-E1 cells subjected to triangular waveform cyclic stretch for 6, 12, 18, and 24 h is shown (Fig. 1). The same area was photographed at each time point except monolayer at 24 h. In the conventional monolayer model, cells aligned perpendicular to the direction of stretch as reported in many previous studies (Kurpinski et al. 2006; Matsugaki et al. 2013; Kim et al. 2020). On the other hand, in the multilayered model, the lower layer cells align in the direction of stretch, whereas the upper layer cells were observed to align in a direction perpendicular to the direction of stretch after 24 h of cyclic stretch. The changes in the perpendicular orientation index *R*_*A*_ are shown for the lower and upper layers of the multilayer and monolayer cells in Fig. 2. The alignment angle distribution used to calculate *R*_*A*_ is shown in supplement (Fig. S3). Monolayer cells aligned in the perpendicular direction to the stretch after 12 h of cyclic stretch and showed an even stronger alignment at 24 h, although *R*_*A*_ was significantly smaller than that with calyculin A (P < 0.001). In multilayer cells, the lower layer cells initially began to align in the direction of stretch and stabilized around 12 h. The upper layer cells, within 12 h, aligned somewhat in the direction of stretch, similar to the lower layer cells, but then began to align in a direction perpendicular to the stretch. At 24 h, both the lower and upper layer cells aligned orthogonally to one another. The response of monolayer cells to cyclic stretch with calyculin A is discussed in Sect. 3.3.

### Time-dependent deformation of upper and lower layer cells under cyclic stretch

**Fig. 3:**
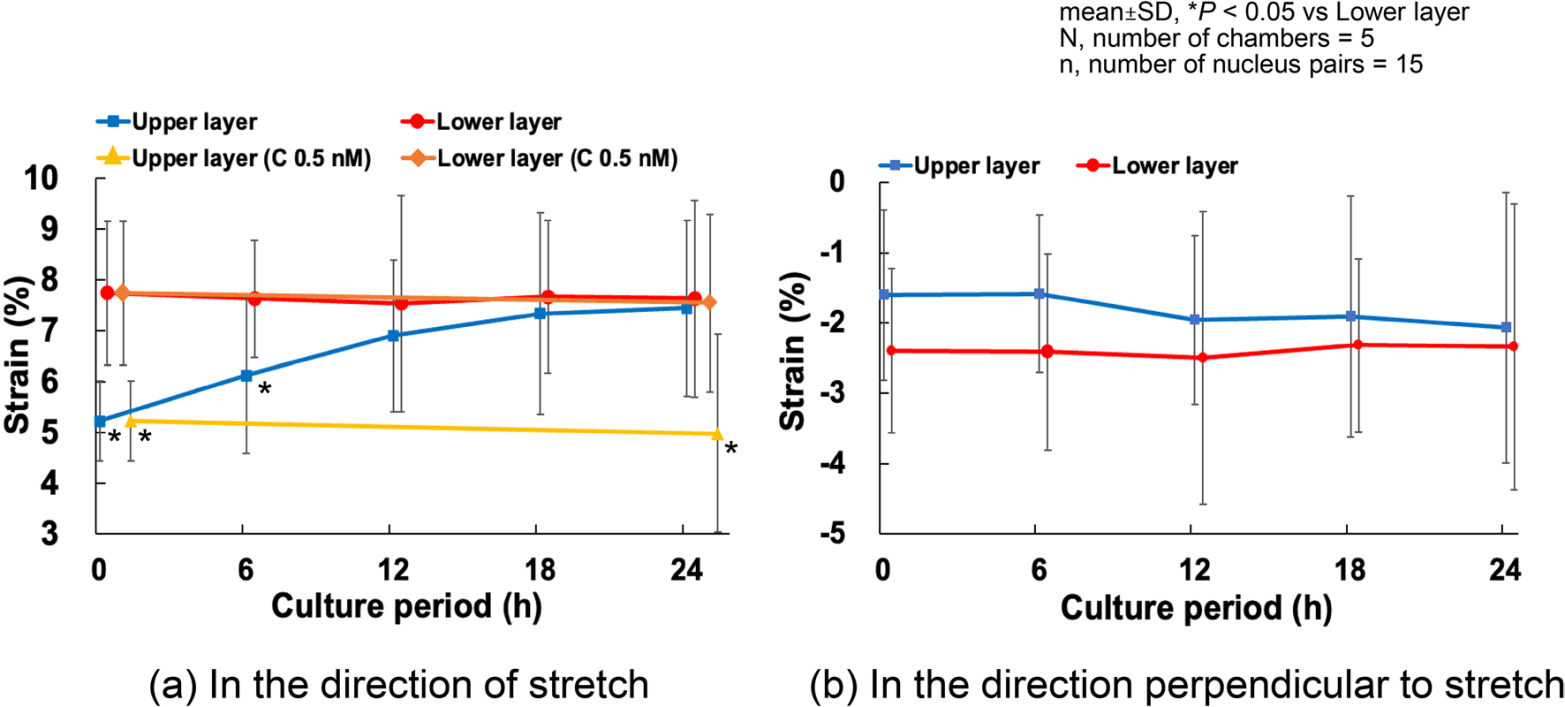
Time course changes of the strain in cells in multilayers subjected to 10% cyclic stretch. C, calyculin A

Cell alignment is closely related to the magnitude of deformation applied to the cells (Kaunas et al. 2005). Therefore, as described in Sect. 2.6, we investigated how cell deformation differs between the upper and lower layers. Strains on cells in both layers were measured at 0, 6, 12, 18, and 24 h of cyclic stretch. The strain transmitted to the lower layer cells was slightly less than 8% in the direction of stretch at all time points. In contrast, the strain in the direction of stretch transmitted to the upper layer cells was significantly lower before cyclic loading (5.22 ± 0.78%) and increased gradually, reaching 6.12 ± 1.53% after 6 h of cyclic loading. By 12 h, the difference between the two layers became insignificant, and after 24 h, the strain on the upper layer cells reached 7.44 ± 1.72% (Fig. 3a). In multilayer cultures treated with calyculin A (0.5 nM), the lower layer cells exhibited similar deformation to untreated multilayers, showing no difference between 0 and 24 h. In contrast, upper layer cells treated with calyculin A did not show the strain increase observed in untreated cells after 24 h of tensile loading, and inter-nuclear strain remaining at 4.97 ± 1.95%. In the direction perpendicular to the tensile axis, the inter-nuclear distance in the lower layer cells consistently showed a compressive strain of approximately 2.4% (Fig. 3b). No significant trends were observed.

### Analysis of actin filaments when intracellular tension is altered

It is well known that changes in intracellular tension influence cellular orientation responses to cyclic stretch. To further investigate this, we analyzed the orientation of MC3T3-E1 monolayer and multilayer cells after 24 h of cyclic stretch under conditions where intracellular tension was decreased with Y-27632 (ROCK-specific inhibitor) and increased with calyculin A (phosphatase inhibitor).

**Fig. 4:**
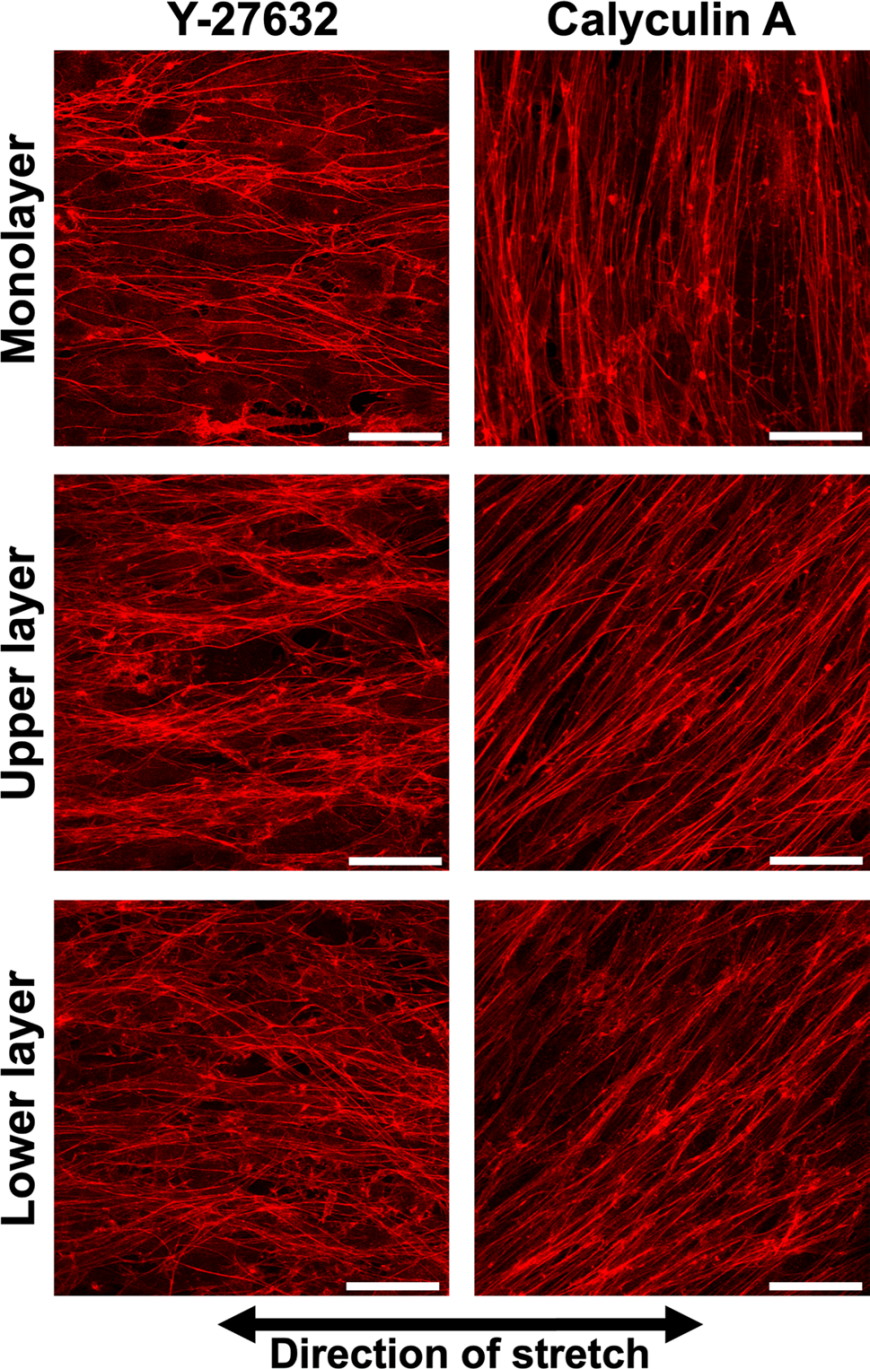
Example of the fluorescent images of actin filaments in MC3T3-E1 cells in monolayer and multilayers treated with Y-27632 or calyculin A and cyclic stretch for 24 h hours. Upper and lower layer in each condition were taken at the same position. Bars = 50 µm

**Fig. 5:**
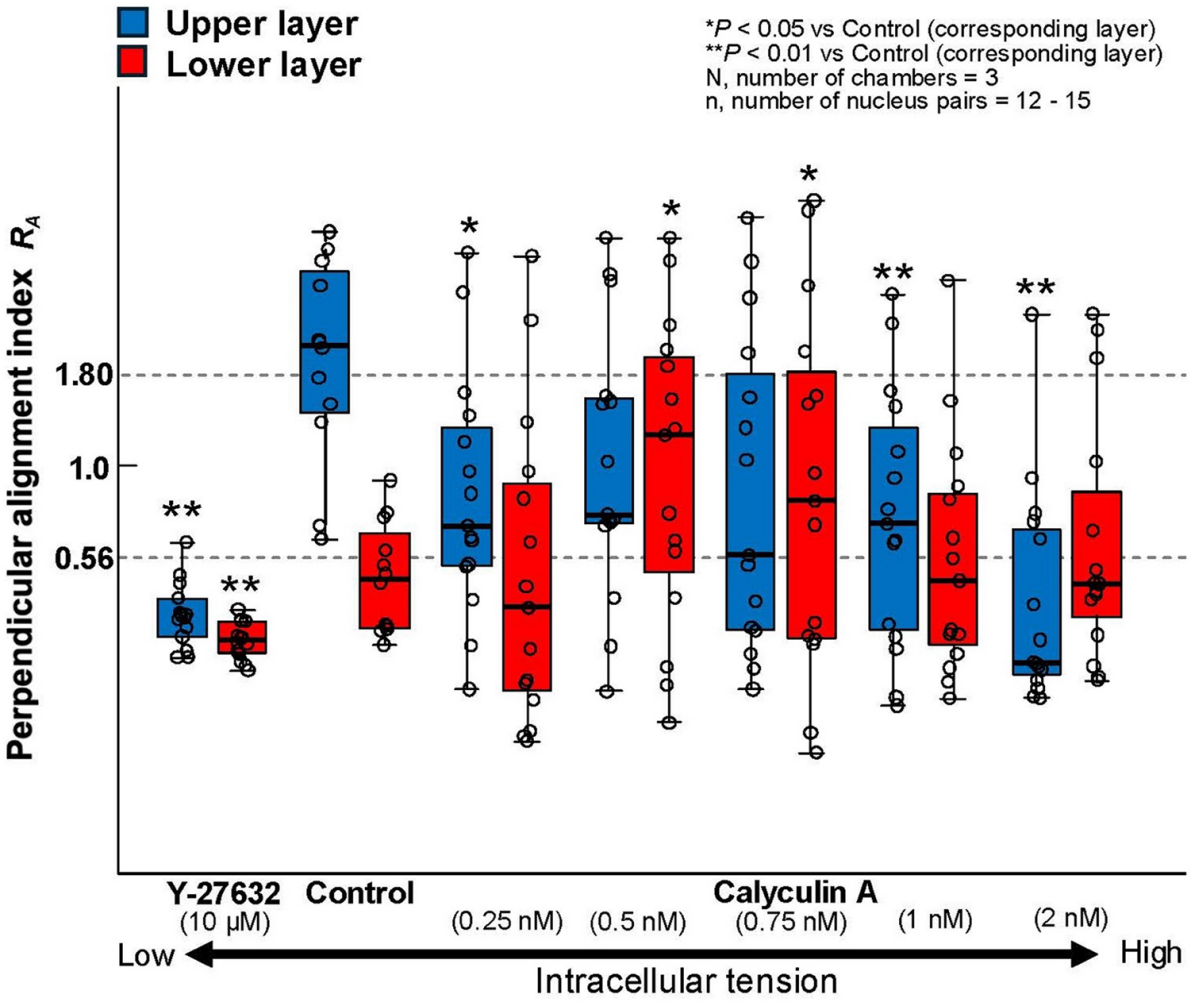
Change in perpendicular alignment index (*R*_*A*_) of multilayer MC3T3-E1 cells exposed to cyclic stretch for 24 h in response to tension manipulation agents (Y-27632 or calyculin A). Statistical differences were compared between the Control and Y-27632 group, and Control and Calyculin A groups

In monolayer cells treated with Y-27632, cells showed AF alignment along the direction of stretch after 24 h of cyclic stretch (Fig. 4). Similarly, in multilayer cells, alignment in the direction of stretch was observed in both the upper and lower layers (Fig. 4). For monolayer cells treated with 0.5 nM calyculin A, AF alignment perpendicular to the direction of stretch occurred faster and more strongly compared to untreated cells as long as judged at the 12- and 24-h time points (Fig. 2 and 4). In multilayer cells, specific alignment was not observed, irrespective of the calyculin A concentration as indicated in wider variation of *R*_A_ (Fig. 5).

### Distribution of focal adhesions in multilayered cells

**Fig. 6:**
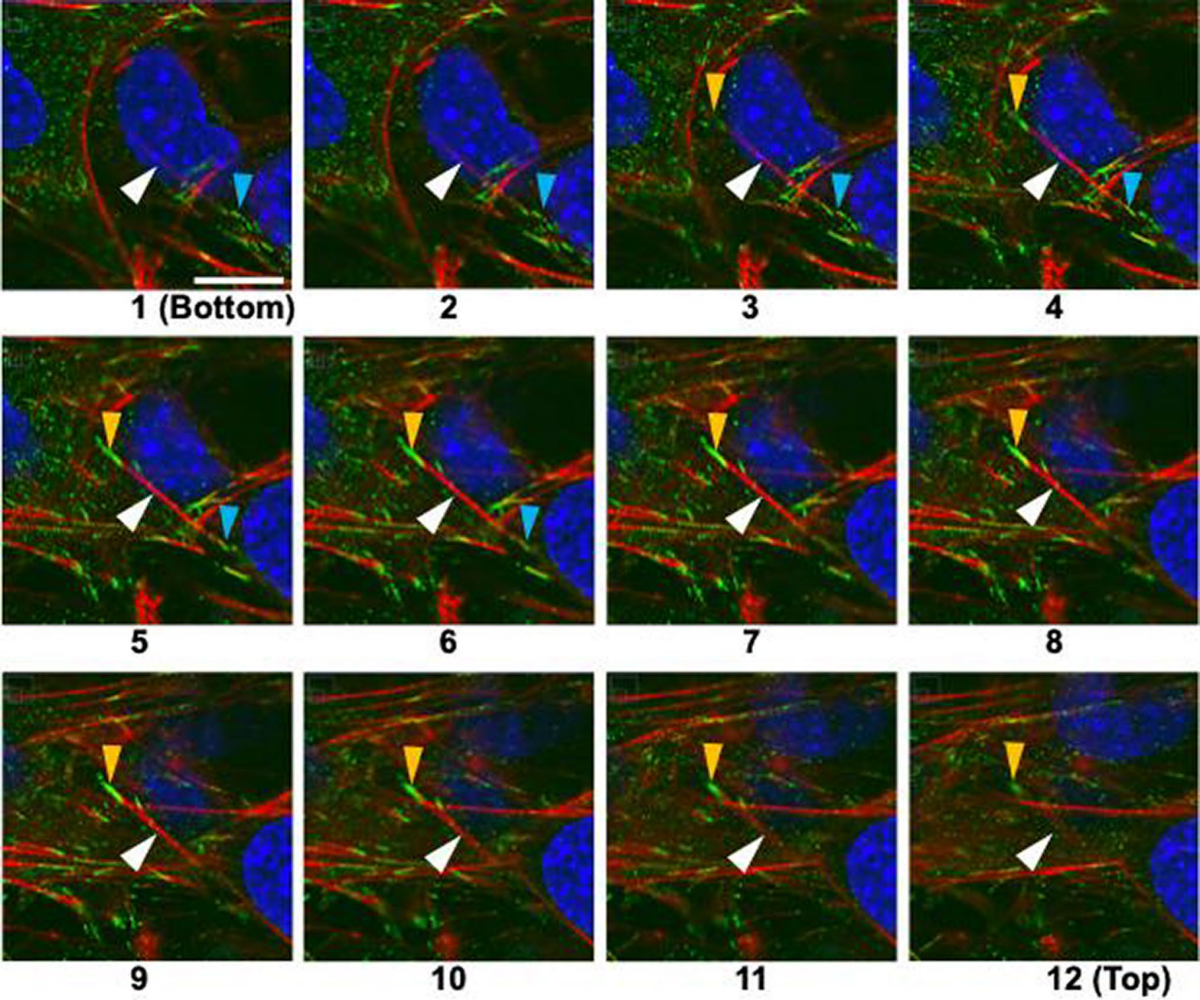
Serial sections of layered MC3T3-E1 cells with a stress fiber (red) connecting upper and lower cells. Red, actin filament (stress fiber); green, vinculin (FA); blue, nucleus. The focus shifts from the substrate surface to the upper layer from Sects. 1 (bottom) to 12 (top). The light blue arrowheads in frames 1 to 6 and yellow ones in frames 3 to 12 indicate FA formed on the ventral and dorsal surface of the lower layer cells, respectively. White arrowheads indicate an actin filament connecting these two FA. Bar = 10 µm applies to all

In monolayer cells, it is known that FA forms only on the basal surface. To examine how FAs are formed between upper and lower layer cells in multilayers, vinculin was stained and observed using a confocal laser microscope. Figure 6 shows fluorescent images of multilayer cells captured at 0.16-μm intervals, where the focus shifts from the lower to the upper layer cells as the number increases.

At Position 1, the FA indicated by the light blue arrowhead begins to appear, followed by the AF indicated by the white arrowhead, which extends toward the upper left of the frame and eventually connects to the FA indicated by the yellow arrowhead. The FA indicated by the light blue arrowhead is observed at Positions 1–6, and the FA indicated by the yellow arrowhead is visible at Positions 3–12. From this, it is suggested that the FA indicated by light blue arrowhead is located near the substrate surface, while the FA indicated by yellow arrowhead is approximately 1.8 μm above it, *i.e.*, on the upper surface of the lower-layer cell.

## Discussion

We found that when cyclic stretch was applied to a multilayer of MC3T3-E1 cells cultured on an elastic substrate, the lower layer cells aligned in the direction of stretch, while the upper layer cells aligned in the direction perpendicular to the stretch. To the best of our knowledge, this is the first study investigating the effects of cyclic stretch on a multilayer cell system, and it is also the first report demonstrating orthogonal alignment of cells in such a configuration. The reasons behind this unique alignment pattern are discussed below.

First, we observed the time-dependent changes in the alignment of the multilayered cells. Initially, the lower layer cells began aligning in the direction of stretch, while the upper layer cells tended to follow this alignment until 12 h after the initiation of cyclic stretch. Subsequently, at 24 h, the lower layer cells remained aligned in the direction of stretch, whereas the upper layer cells demonstrated alignment perpendicular to the direction of stretch. In contrast, for monolayer cells, perpendicular alignment was observed within 12 h of cyclic stretch. These results suggest that, for the upper layer cells, strain induced by substrate deformation was not transmitted initially but began affecting the upper layer cells after a certain period, leading to their alignment in the direction perpendicular to the direction of stretch.

To investigate this hypothesis, we measured the strain transmitted to both the upper and lower cell layers during substrate deformation. The results revealed that the strain in the upper cell layer increased from 0 to 12 h after the onset of tensile loading, reaching approximately 7.4% at 12 h. Beyond 12 h, the strain applied to the upper layer remained relatively constant, suggesting a steady transmission of strain. These indicate that once the upper layer began experiencing strain, their response became similar to that of monolayer cells within 12 h.

Next, we consider why the lower layer cells aligned in the direction of stretch. There have been several studies that reported cell alignment in the direction of cyclic stretch. For example, Matsumoto et al. (1996) demonstrated that macrophages aligned in the direction of stretch under cyclic stretch. Similarly, there are reports that administering Y-27632 to bovine aortic endothelial cells (BAECs), which reduces tension in the actin cytoskeleton, induces alignment in the direction of stretch (Kaunas et al. 2005). Furthermore, when cyclic stretch was applied to NIH3T3 fibroblasts derived from mouse embryos treated with Y-27632, the cells aligned in the direction of stretch (Nagayama and Fukuei, 2019). Additionally, human osteosarcoma cells (U2OS) and human mesenchymal stem cells (hMSC) cultured on soft collagen gels have been reported to align in the direction of stretch (Tondon and Kaunas 2014). It is also well known that cells cultured on soft substrates exhibit reduced intracellular tension (Califano et al., 2010). These results suggest that 1) cells with significant cytoskeletal tension tend to align perpendicular to the direction of stretch or in the direction of minimal strain (Wang et al. 1995, 2001; Takemasa et al. 1998; Nagayama et al. 2012; Livne et al. 2014), in contrast, 2) cells with low cytoskeletal tension, either due to intrinsic properties or chemical modulation, tend to align in the direction of stretch. In our multilayered system, the lower layer cells might experience a reduction in intracellular tension due to the presence of upper layer cells, and thus, they might align in the direction of stretch.

To confirm this, we modulated the cytoskeletal tension of multilayered MC3T3-E1 cells using pharmacological agents and observed their responses under cyclic stretch (Figs. 4 and 5). When cytoskeletal tension was reduced, both the upper and lower layers aligned in the direction of stretch, as observed in MC3T3-E1 monolayer with Y-27632. When intracellular tension was increased using calyculin A, we anticipated that both layers would align perpendicular to the direction of stretch. However, the cells exhibited a disorganized alignment, showing no specific orientation. The cause of this remains unclear, but we propose a hypothesis that the formation of FA on both the ventral and dorsal sides of the lower layer cells might make the intracellular tension non-uniform, which may lead to the disorganized alignment.

**Fig. 7:**
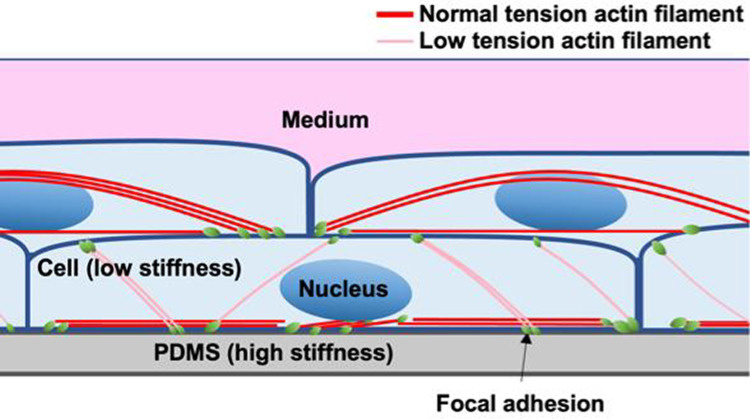
Schematic diagram of stratified cells. FA forms both on the ventral and dorsal side in the lower layer cells. The AFs connecting FA on the ventral side may develop sufficient tension because the substrate (PDMS) is stiff, while the tension in the AFs connecting FA on the ventral and dorsal side might not high enough, because one of their ends are connected to the cells that is not stiff. In case of the upper layer cells, FA forms only on the ventral side, and the tension in the AFs connecting these ventral FA might not so high

To investigate this, vinculin was stained and observed using fluorescence microscopy. Vinculin is known to be present in both cell–substrate adhesions and cell–cell adhesions (Bays and DeMali 2017), but since the vinculin observed in this study was connected only to the AF from below, it is considered to be derived from FA. We currently hypothesize that this is because the cells produce collagen between the upper and lower layers. Thus, we found that in multilayered cells, FA appear not only on the lower side, *i.e.*, ventral side, but also on the upper side, *i.e.*, dorsal side of the lower layer cells. On the other hand, it is well known that FA in monolayer cultures formed only on the ventral side, with AFs bridging between these FA. The Young's modulus of the substrate (PDMS) is approximately 2 MPa (Lee et al. 2004), much higher than the cellular Young's modulus of tens of kPa (Nagayama et al. 2006). Stiff substrate allows sufficient tension to develop in the AFs in the monolayer cells. In multilayered cells, however, AFs also form in the lower layer cells connecting FA on the dorsal and ventral side, likely limiting the development of sufficient tension (Fig. 7). This reduction in intracellular tension in the lower layer cells could phenomenologically explain their alignment in the direction of stretch. In the lower layer cells, AFs also form connecting two FA on the ventral side. These AFs may develop sufficient tension. Thus, tension in the AFs in the lower layer cells with FA both on ventral and dorsal sides might not uniform.

**Fig. 8:**
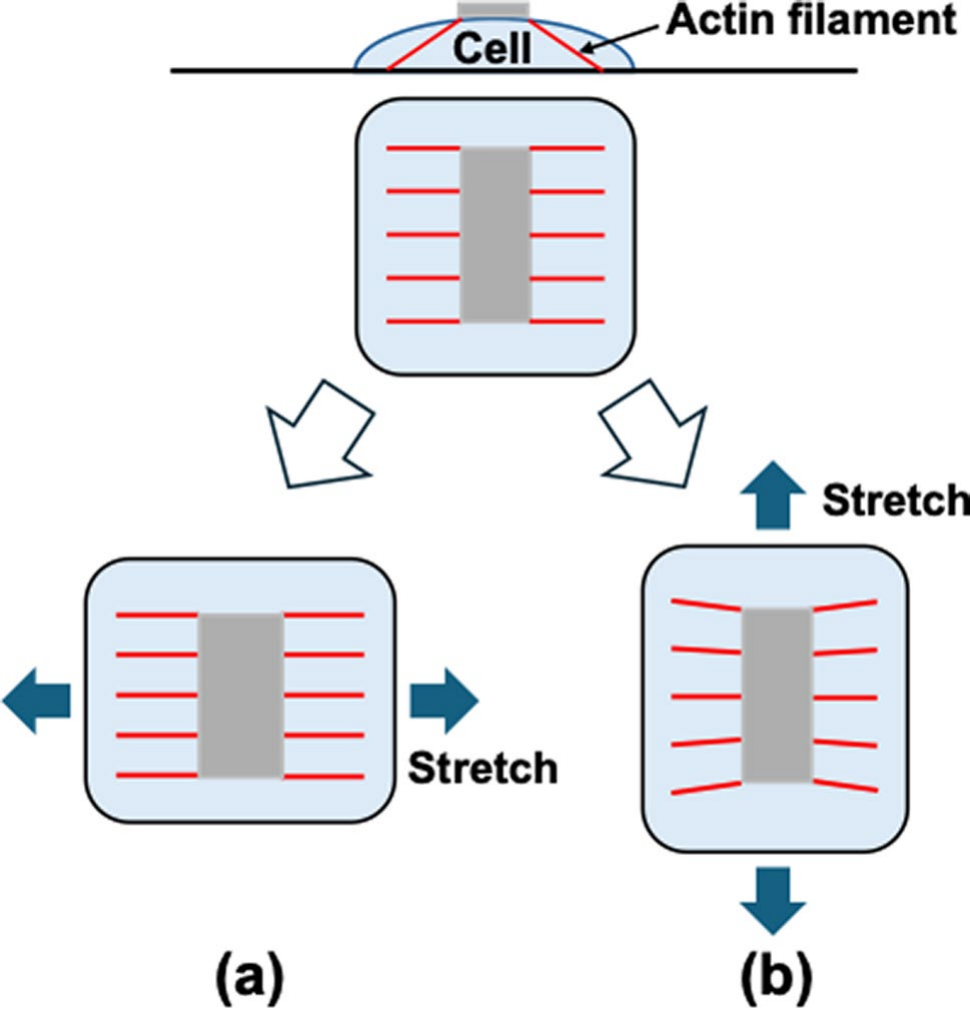
Schematic explanation of the effect of AF orientation on the transmission of the substrate deformation to the upper surface of cells. When AFs are aligned in the direction of stretch (a), the deformation of the substrate may transmitted directly to the upper surface of the cell, while when they are aligned perpendicular to the direction of stretch (b), the substrate deformation seldom transmitted to the upper surface. This may indicate the random orientation of the AFs may reduce the deformation of the upper surface significantly

On the other hand, the upper layer cells are connected to the dorsal surface of the lower layer cells, and the AFs connecting the dorsal and ventral side of the lower layer cells align in the direction of the stretch. Therefore, the deformation of the substrate may transmit directly to the ventral surface of the upper layer cells (Fig. 8a), as observed in the upper layer cells without calyculin A at 24 h (Fig. 3a), which may cause the upper layer cells to align in a direction perpendicular to the cyclic stretch. However, when calyculin A was applied to increase intracellular tension, the alignment of AFs in the stretch direction disappeared in the lower layer cells. This may cause insufficient transmission of the substrate deformation to the upper layer cells (Fig. 8b) as observed in the upper layer with calyculin A (Fig. 3a), leading to their random alignment.

In summary, we investigated the morphological responses of multilayered osteoblast-like MC3T3-E1 cells under cyclic stretch. The lower layer cells, with reduced tension due to the presence of the upper layer cells, aligned in the direction of stretch. Their AFs were oriented along the direction of stretch, allowing substrate deformation to be directly transmitted to the FA on the dorsal surface. The upper layer cells, forming FA only on their ventral surface, likely aligned perpendicular to the direction of stretch, similar to monolayer cells.

In future studies, we plan to induce FA formation on the upper surface of monolayer cells and examine their alignment. Additionally, using cells isolated from transgenic mice expressing FRET-based tension sensors recently established in our laboratory (Wang et al. 2023), we aim to directly observe changes in intracellular tension. These approaches will help clarify the mechanisms underlying the orthogonal alignment between the upper and lower layers of multilayered MC3T3-E1 cells.

## Supplementary Information

Below is the link to the electronic supplementary material.

Supplementary file1 (DOCX 1482 KB)

## Data Availability

No datasets were generated or analysed during the current study.
