# Supplementary material for: Orthogonal alignment of multilayered MC3T3-E1 cells induced by cyclic stretch
Source: Biomech Model Mechanobiol. 2025 Jul 2;24(5):1501–11. doi: 10.1007/s10237-025-01978-z (PMC12454628; doi:10.1007/s10237-025-01978-z)
Supplement: Supplementary file 1 — Supplementary file1 (DOCX 1482 KB) [file 10237_2025_1978_MOESM1_ESM.docx]

**Supplement**

**(c)**

**Fig. S1** Schema (a) and photograph (b) of benchmarks used for strain measurement of silicone membrane, and measured strain of silicone membrane exposed to triangular wave (c)

**Fig. S2** Method for measuring intercellular strain. Bars = 20 µm

**Fig. S3** The alignment angle distribution of actin filaments in layered MC3T3-E1 cells exposed to cyclic stretch. (N = 6, n = 30; N = number of chambers, n = number of points in each group, **P* < 0.05, ***P* < 0.01 vs 0h in each layer)

**Table S1** Strains in measured areas of silicone membrane exposed to triangular wave. Mean ± SD (calculated for n); *N*, number of chambers = 2; *n*, number of marks = 4.

| Area | Longitudinal strain (%) | Lateral strain (%) |
| --- | --- | --- |
| 1 | 8.89 ± 1.02 | -4.36 ± 0.34 |
| 2 | 8.39 ± 0.60 | -4.50 ± 0.49 |
| 3 | 8.44 ± 0.64 | -4.06 ± 0.48 |
| 4 | 8.01 ± 0.57 | -4.46 ± 0.36 |

**Table S2** Direction with minimum strain amplitude. Mean ± SD (calculated for n); *N*, number of chambers = 2; *n*, number of marks = 4.

| Area | Minimum strain direction (°) |
| --- | --- |
| 1 | 59.3 ± 2.00 |
| 2 | 58.5 ± 2.51 |
| 3 | 59.9 ± 1.79 |
| 4 | 57.8 ± 1.26 |
